# Efficacy and Safety Analysis of Immune Checkpoint Inhibitors plus Angiogenesis Inhibitors for the Treatment of Advanced Driver-negative NSCLC in Elderly Patients: A Retrospective Study

**DOI:** 10.7150/jca.83719

**Published:** 2023-06-04

**Authors:** Jian Zhang, Zhonghua Zou, Jie Tan, Jianping Shi, Hui Yang, Hao Wang, Jundong Zhou, Jing Xue

**Affiliations:** 1 Department of Radiation Oncology, The Affiliated Suzhou Hospital of Nanjing Medical University, Suzhou Municipal Hospital, Gusu School, Nanjing Medical University, Suzhou 215001, China.; 2 Department of Medical Oncology, The Affiliated Suzhou Hospital of Nanjing Medical University, Suzhou Municipal Hospital, Gusu School, Nanjing Medical University, Suzhou 215001, China.; 3 Department of Radiation Oncology, The First Affiliated Hospital of Anhui Medical University, Hefei 230031, China.

**Keywords:** immune checkpoint inhibitors, angiogenesis inhibitors, non-small cell lung cancer, elderly patient, retrospective

## Abstract

**Background and Objective:** Immune checkpoint inhibitors (ICIs) combined with angiogenesis inhibitors may have synergistic effects in elderly patients with advanced driver-negative NSCLC, but its true efficacy remains unclear. In addition, chemotherapy tolerance in elderly NSCLC patients is poor, and the precise identification of the population that may benefit from ICIs combined with angiogenesis inhibitors is also the focus of current research.

**Methods:** We retrospectively compared the efficacy and safety of ICIs combined with or without antiangiogenic agents in elderly patients with advanced driver-gene negative NSCLC ≥65 years of age in the Cancer Center of Suzhou Hospital Affiliated to Nanjing Medical University. The primary endpoint was PFS. Secondary endpoints were OS, ORR, and immune-related adverse events (irAEs).

**Results:** A total of 36 patients in the IA group (immune checkpoint inhibitors plus angiogenesis inhibitors group) and 43 patients in the NIA group (immune checkpoint inhibitors without angiogenesis inhibitors group) were enrolled in the study between January 1, 2019 and December 31, 2021. The median follow-up time for patients in the IA group and NIA group was 18.2 months (95%CI: 14 - 22.5 months) and 21.4 months (95%CI: 16.7 -26.1 months), respectively. The median PFS and median OS were longer in the IA group compared to the NIA group (8.1 months vs 5.3 months; HR for PFS: 0.778, 95%CI: 0.474-1.276, P=0.32; NA vs 30.9 months; HR for OS: 0.795, 95%CI: 0.396-1.595, P=0.519). There were no significant differences in median PFS and median OS between the two groups. Subgroup analysis showed that patients in the IA group had significantly longer PFS in the subgroup with PD-L1 expression ≥50% (*P*=0.017), and the association between different groups and disease progression was still different in the two subgroups (*P for interaction* = 0.002). There was no significant difference in ORR between the two groups (23.3% vs 30.5%, *P*=0.465). The incidence of irAEs in the IA group was lower than that in the NIA group (39.5% vs 19.4%, *P*=0.05), and the cumulative incidence of treatment interruptions due to irAEs was significantly reduced (*P*=0.045).

**Conclusion:** In elderly patients with advanced driver-negative NSCLC, the addition of antiangiogenic agents to ICIs therapy did not provide significant clinical benefit, but the incidence of irAEs and treatment interruptions due to irAEs was significantly reduced. In the subgroup analysis, we found that the clinical benefit of this combination therapy was observed in patients with PD-L1 expression ≥50%, which warrants further exploration.

## Introduction

As China's aging population worsens, the proportion of elderly patients with new lung cancer has increased, and most of them have advanced NSCLC 1. The classic first-line treatment regimen for advanced driver-negative NSCLC is based on platinum-based doublet-chemotherapy 2, while there is no consensus on the treatment of elderly patients. According to current studies, the treatment of advanced NSCLC in elderly patients with good physical condition is advocated to be based on chemotherapy 3-5, but this treatment philosophy has changed with the advent of immune checkpoint inhibitors (ICIs). KEYNOTE-024 6 and KEYNOTE-042 7 subgroup analyses found that immunotherapy alone significantly improved median OS compared to chemotherapy in elderly patients with PD-L1 ≥ 50% in advanced driver-negative NSCLC. Subgroup analysis of the KEYNOTE-189 study 8, 9 showed that pembrolizumab combined with chemotherapy could further improve median OS in elderly patients. In addition, the Phase III Camel clinical study 10 and KEYNOTE-407 Chinese cohort study subgroup analysis 11 also showed that ICIs combined with chemotherapy could prolong median PFS in elderly patients (HR=0.57; HR=0.45, respectively). Immunotherapy is an emerging antitumor modality in recent years, and its efficacy in elderly patients indicates the direction for us to find treatment strategies to improve the prognosis of elderly patients with advanced NSCLC.

IMpower150 12, a Phase III randomized controlled study in patients with advanced non-squamous NSCLC, found that adding atezolizumab to bevacizumab+ carboplatin+ paclitaxel significantly prolonged median PFS (8.3 months vs. 6.8 months, HR=0.62, *P*<0.001) and median OS (19.2 months vs. 14.7 months, HR=0.78, *P*=0.02) without increasing the incidence of grade ≥3 adverse events. Further analysis of IMpower150's final overall survival 13 found that four drug combinations could still significantly prolong median OS in patients compared to bevacizumab+ doublet-chemotherapy (19.5 months vs 14.7 months, HR=0.80). Another Phase Ib clinical study (NCT03628521) in which anlotinib was added to sintilimab as first-line treatment for advanced or metastatic NSCLC with driver-negative 14 showed an ORR of 72.7%, a median PFS of 15 months, a 12-month progression-free survival rate of 71.4%, and ≥ grade 3 adverse events of 54.5%. These studies suggest that ICIs combined with antiangiogenic drugs may have a synergistic effect, but since less than 50% of patients were elderly, it is unclear whether the findings can be generalized to elderly patients. In addition, one study 15 found that anti-angiogenic drugs are well tolerated in elderly patients with advanced NSCLC, suggesting that ICIs combined with anti-angiogenic drugs may be a better immune combination.

Therefore, we aim to explore the clinical significance of ICIs combined with anti-angiogenesis drugs in elderly patients with advanced driver-negative NSCLC using real-world data.

## Materials and Methods

### Patients

We reviewed the medical records of all lung cancer patients at the Cancer Diagnosis and Treatment Center of Suzhou Hospital Affiliated to Nanjing Medical University from January 1, 2019 to December 31, 2021. A total of 79 patients met the following inclusion and exclusion criteria: 1. Age ≥ 65 years; 2. Stage IV according to the AJCC Cancer Grading Manual (8th edition); 3. NSCLC confirmed by histopathology; 4. No driver gene mutation; 5. Received at least 2 immunotherapy courses; 6. Expected survival > 3 months; 7. No concurrent malignancies; 8. Not participating in clinical trials; 9. Basic normal function of important organs; 10. Signed informed consent. All enrolled patients were followed up until August 1, 2022. All data were double-checked, and patients who had no outcome event at the end of the study were critically rechecked. Patients were divided into Immune checkpoint inhibitors plus angiogenesis inhibitors group (IA group) and Immune checkpoint inhibitors without angiogenesis inhibitors group (NIA group).

### Study design

PFS, OS, ORR, covariates, and immune-related adverse events (irAEs) data were collected through electronic medical records and follow-up. The primary endpoint of the study was PFS, and the secondary endpoints were OS, ORR, and irAEs. PFS was defined as the time from the start of the first immunotherapy to disease progression or death. OS was defined as the time from the start of the first immunotherapy to death from any cause. ORR refers to the sum of complete response (CR) and partial response (PR) to treatment. Endpoints and irAEs were assessed in strict accordance with RECIST v.1.1 and CTCAE v.5.0, respectively.

### Statistical analysis

Continuous variables were expressed as mean ± SD, and independent samples t-test was used if the two groups were normally distributed and had homogeneous variance, otherwise Mann-Whitney U test was used. The categorical variables were expressed as percentages, usually using the Χ^2^ test or Fisher's exact probability method. Kaplan-Meier was used to analyze PFS and OS, and Log-rank was used to compare groups. To avoid confounding factors, factors that were clinically significantly associated with prognosis and those with *P*<0.1 in the univariate analysis were included in the COX regression analysis. The cumulative incidence of treatment discontinuation due to adverse events was tested by Fine-Gray test, and disease progression or death was used as competing events. SPSS v.19.0 was used for data analysis and R 4.2.2 (Murray Hill, NJ, USA) was used to draw the forest plot and cumulative incidence curve.* P*<0.05 indicates that the difference between groups was statistically significant.

## Results

### Patient characteristics

**Figure 1:**
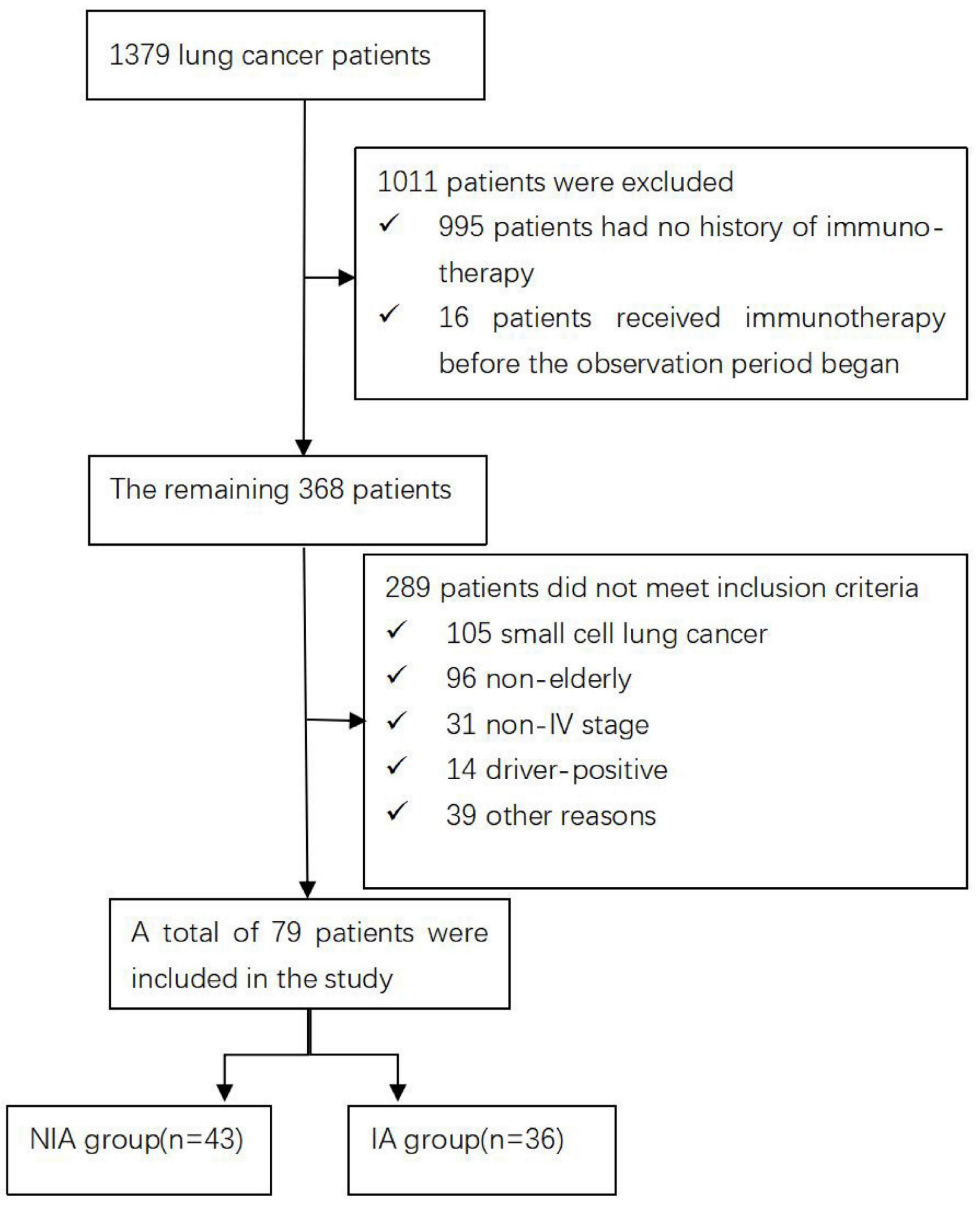
Flow chart

A total of 1379 lung cancer patients were collected during the observation period. 1011 were excluded because immunotherapy records were not available or were not first used, the remaining 289 were not included in the study because they did not meet the inclusion criteria, and 79 were finally included in the follow-up evaluation and analysis of the current study (Figure 1). Of these, 43 were included in the NIA group and 36 in the IA group, and the baseline characteristics of patients in both groups are summarized in Table 1. The median age was slightly lower in the IA group compared to the NIA group (69.7 years vs. 69.9 years). Squamous cell carcinoma and less than 3 metastatic lesions were slightly more common in the NIA group and less common in the IA group. The number of unmeasured or PD-L1 <1%, PD-L1 1%~50% and PD-L1≥50% in the IA group was 7 (19.4%), 9 (25%) and 20 (55.6%), respectively. The number of patients with brain or bone metastases before initiation of immunotherapy was 24 (55.8%) and 18 (50%), respectively. Smoking, male, ECOG ≤1 score, first-line treatment, no combination radiotherapy, and combination chemotherapy were more frequent in both groups. Except for the uneven distribution of combined chemotherapy and chemotherapy frequency (Table S1) between the two groups, there were no significant statistical differences in other characteristics, and there was no large selection bias. In addition, the tumor burden of all patients is shown in Table S2.

### Progression-free survival

**Figure 2:**
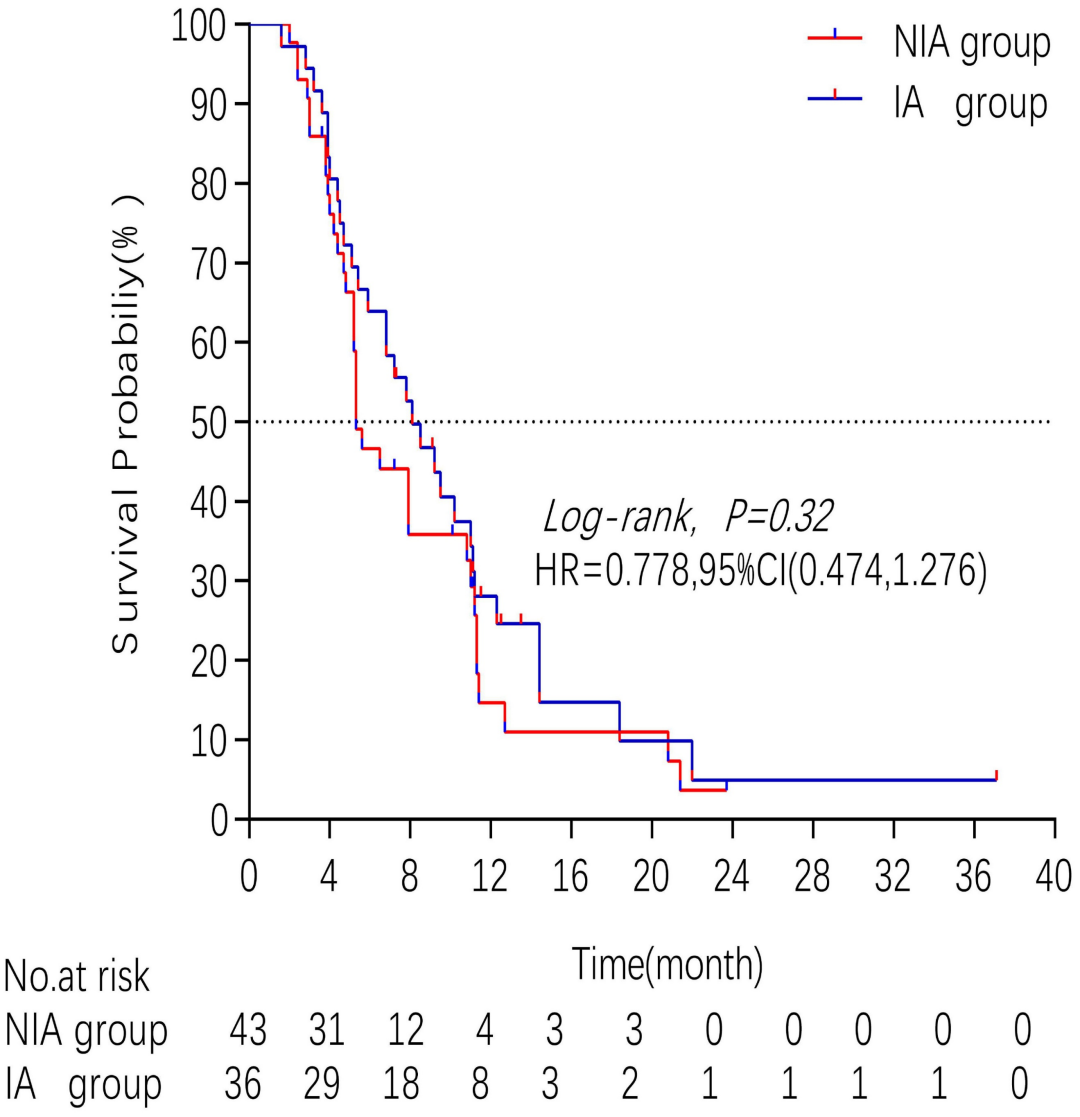
Kaplan - Meier curves comparison of patients on progression-free survival

**Figure 3:**
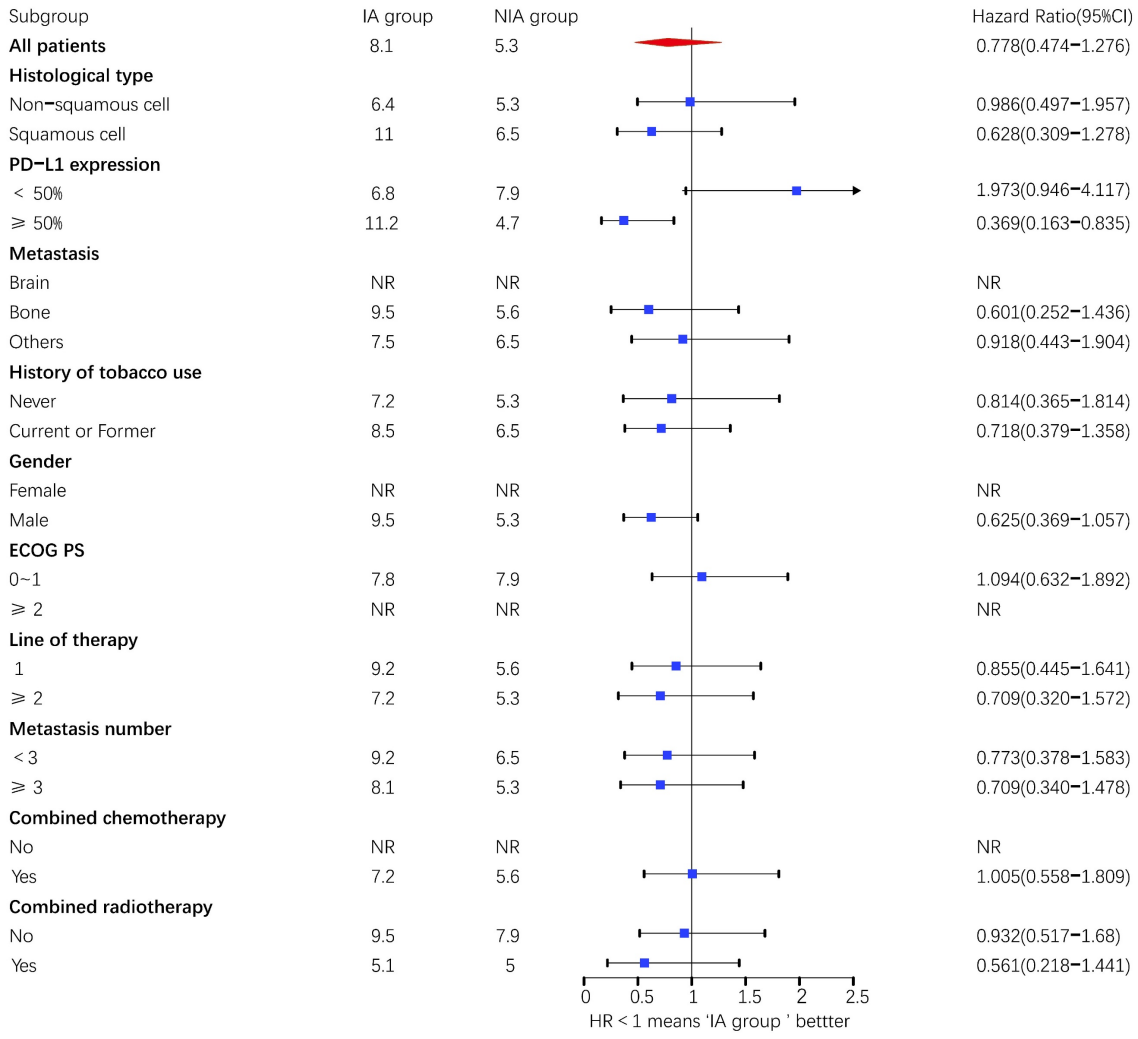
Subgroup analysis of patients on progression-free survival. Upper value above 2.5 in the 95% confidence interval are indicated by the arrow.

**Figure 4:**
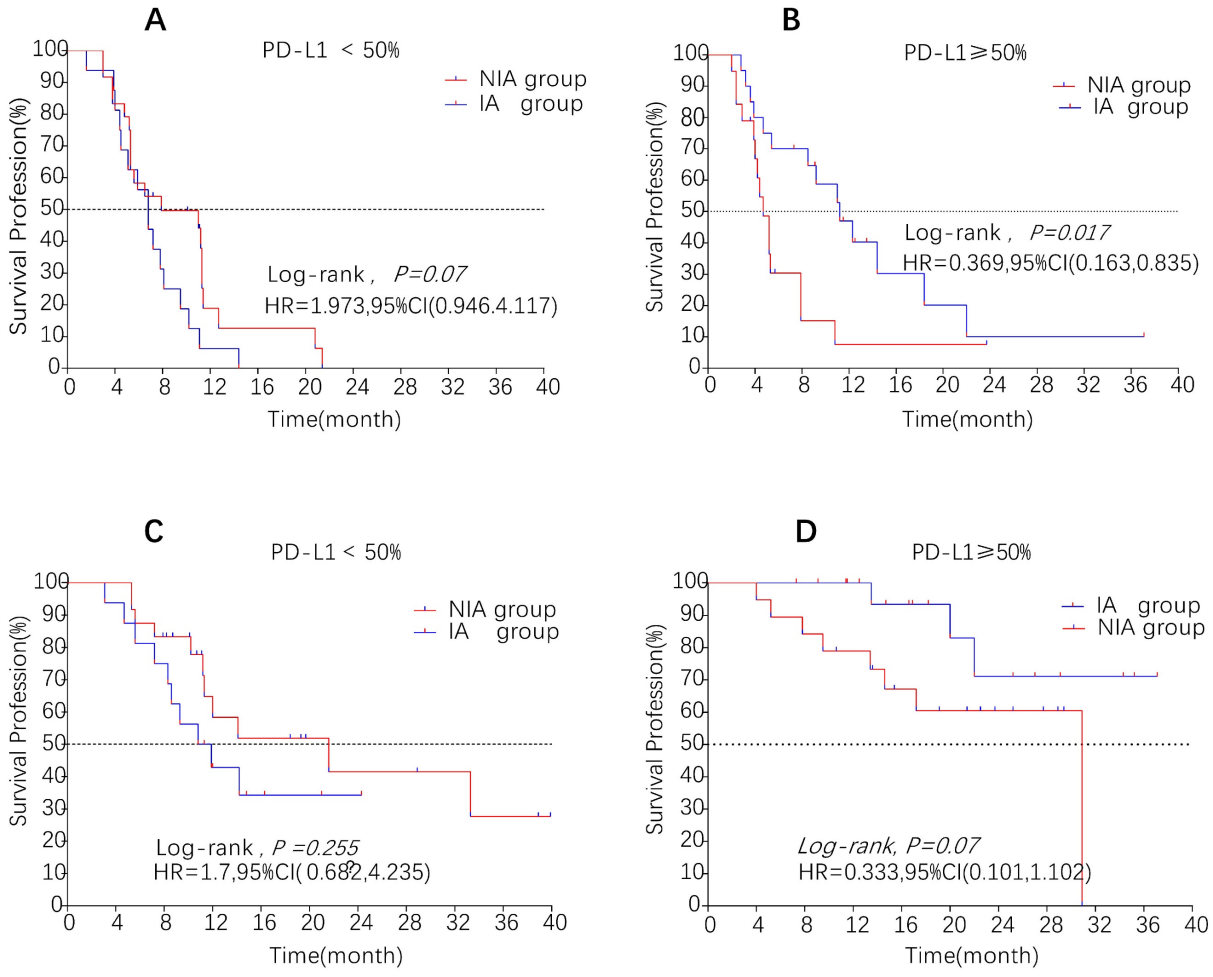
Survival curves for patients in the PD-L1 expression subgroup. A: Progression-free survival curve in PD-L1<50% subgroup; B: Progression-free survival curve in PD-L1≥50% subgroup; C: Overall survival curve in PD-L1<50% subgroup; D: Overall survival curve in PD-L1≥50% subgroup

A total of 65 patients (82.3%) had disease progression, 14 patients were censored (17.7%, 3 lost visits and 11 truncated), of which 35 patients (44.3%) showed disease progression and 8 patients (10.1%) were lost in the NIA group and 30 patients (38%) showed disease progression and 6 patients (7.6%) were lost in the IA group. Median PFS was longer in the IA group than in the NIA group (8.3 months vs. 5.1 months), but the Log-rank test showed that the difference between the two groups was still not statistically significant (HR: 0.778, 95%CI: 0.474-1.276, *P*=0.32) (Figure 2). No significant prognostic factors were found by univariate analysis (Table 2). Subgroup analysis (Figure 3) found that the median PFS of patients in the IA group was longer than in the NIA group except for the subgroup of PD-L1 < 50% and ECOG status 0-1, but this difference was statistically significant only in the subgroup of PD-L1≥50% (Figure 4). In the PD-L1≥50% subgroup, the risk of disease progression in the IA group was 0.631 times lower than that in the NIA group, which was statistically significant (*P*=0.017), and the association between different groups and disease progression was still different in the two subgroups (*P for interaction* = 0.002) (Table 3). In addition, the details of radiotherapy and chemotherapy in patients received combined radiotherapy during PFS period were shown in Table S3.

### Overall survival

**Figure 5:**
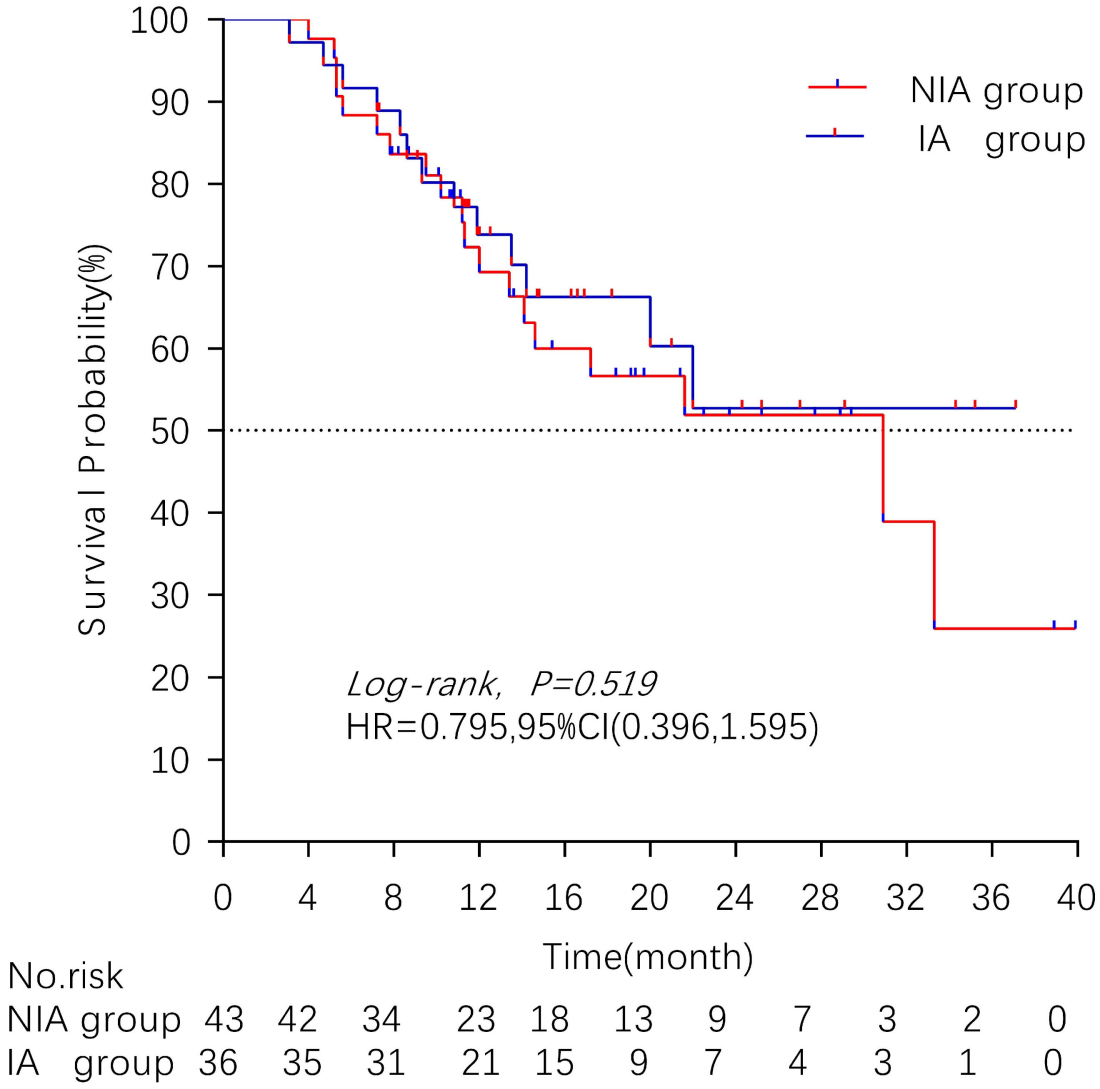
Kaplan-Meier curves comparison of patients on overall survival

**Figure 6:**
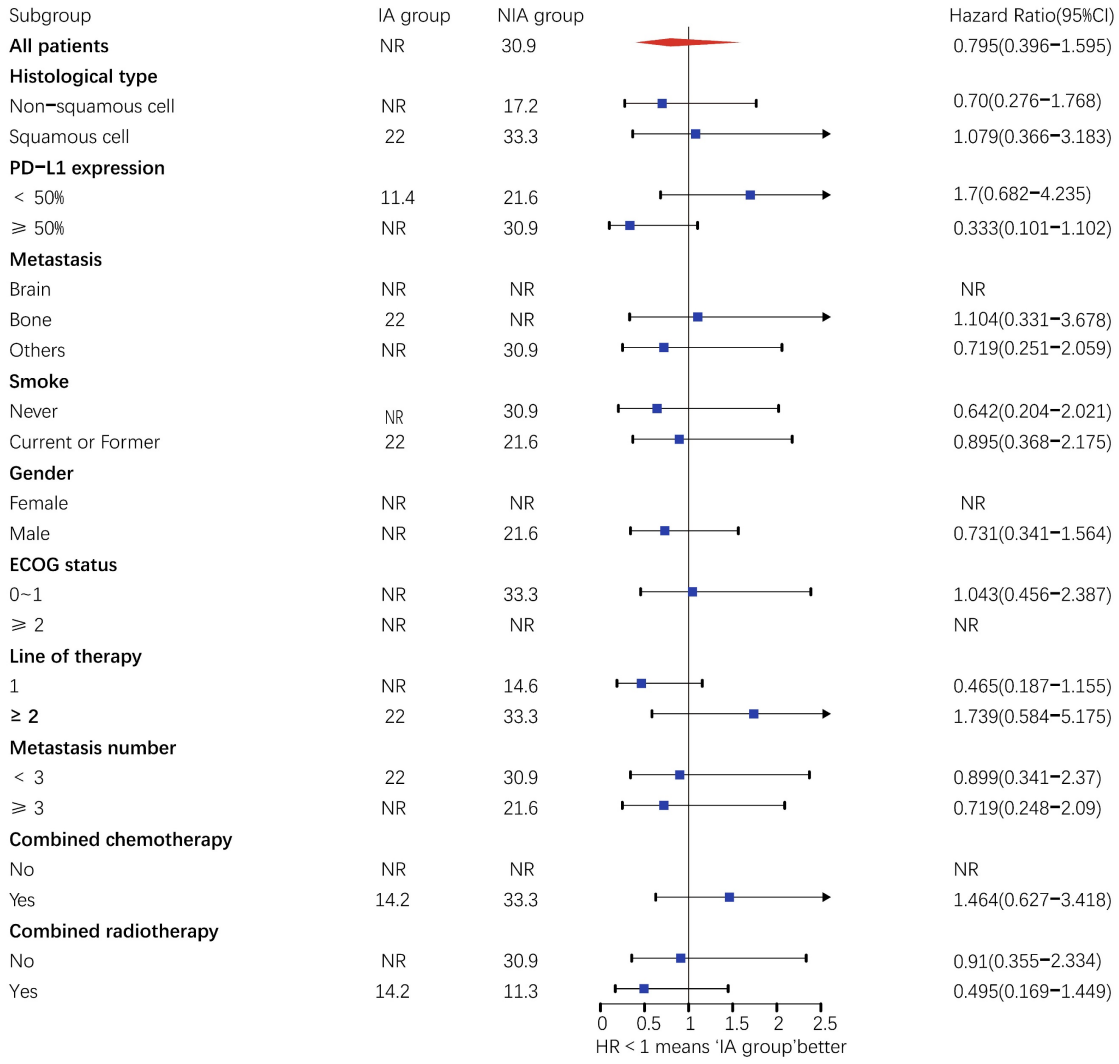
Subgroup analysis of patients on overall survival. Upper value above 2.5 in the 95% confidence interval are indicated by the arrow.

There were 32 (40.5%) deaths and 47 censors (59.5%, 1 lost visit and 46 truncated) in the total population, including 19 (24.1%) deaths and 24 (30.4%) censors in the NIA group and 13 (16.5%) deaths and 23 (29.1%) deletions in the IA group. The median follow-up time for patients in the IA group was 18.2 months (95% CI: 14 months-22.5 months) compared to 21.4 months (95% CI: 16.7 months-26.1 months) in the NIA group, and the log-rank test showed no statistically significant difference in follow-up time between the two groups (Χ^2^=0.882, *P*=0.348). The median OS of patients in the IA group was not reached, and the median OS of patients in the NIA group was 30.9 months, and further log-rank testing revealed that the difference between the two groups was not statistically significant (HR: 0.795, 95%CI: 0.396-1.595, *P*=0.519) (Figure 5). Univariate analysis (Table 4) showed that PD-L1 expression, combination chemotherapy, and combination radiotherapy were associated with prognosis. The metastatic site, treatment line, ECOG, and pathological type that were clinically related to prognosis were included in the multivariate analysis, and only combination with radiotherapy had clinical significance (*P*=0.01), suggesting that combination with radiotherapy was an independent factor related to patient prognosis. Further subgroup analysis did not identify clinically significant subgroups (Figure 6).

### Objective response rate

Throughout the observation period, the NIA group showed 0 CR (0.0%), 10 PR (23.3%), 15 stable diseases (SD, 34.9%), 15 disease progression (PD, 34.9%) and 3 deletions (7%); whereas the IA group showed 1 CR (2.7%), 9 PR (27.8%), 13 SD (36.1), and 11 PD (30.6%), with no statistical difference in ORR between the two groups (23.3% vs. 30.5%, *P*=0.465) (Table 5A). Twenty-eight patients (35.4%) had the time from the best response to disease progression of more than 6 months, and 6 patients (7.6%) had the time of more than 12 months. In addition, among those with PD-L1 ≥50%, ORR increased slightly in the IA group (35% vs 15.8%, *P*=0.17), which still did not appear statistically significant (Table 5B).

### Safety

**Figure 7:**
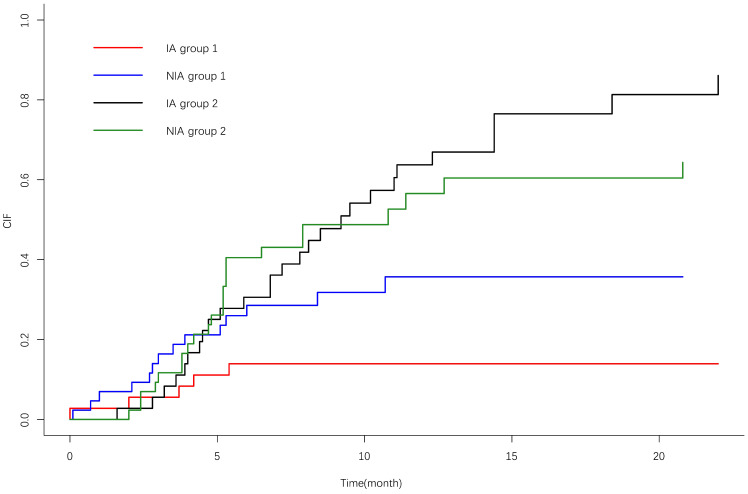
Cumulative incidence curve of treatment interruption due to immune-related adverse events. 1: Treatment interruption due to adverse effects; 2: Competitive risk events: disease progression or death due to lung cancer.

IrAEs occurred in 17 patients (39.5%) in the NIA group and 7 patients (19.4%) in the IA group, and the most common irAEs was pneumonia. There was no significant difference in the incidence of irAEs between the two groups (*P*=0.05). Grade ≥3 irAEs occurred in 9 patients (20.9%) in the study group and 3 patients (8.3%) in the control group, and the most common irAEs was pneumonia (Table 6). A total of 21 patients (26.6%) discontinued treatment due to irAEs, 7 patients (8.9%) discontinued treatment for ≥6 months, 4 patients (5.1%) discontinued treatment for ≥12 months, and 4 patients (5.1%) died due to irAEs. The cumulative incidence of treatment interruptions due to irAEs was significantly lower in the IA group than in the NIA group (*P*=0.045) (Figure 7), and the cumulative incidence curve showed that treatment interruptions due to irAEs occurred slightly earlier in the IA group than in the NIA group. Antiangiogenic drug-related AEs occurred in 22 patients (61.1%) in the IA group, ≥3 grade AEs were 5 patients (13.9%), and the most common AEs was hypertension. Compared to the NIA group, there was no significant difference (*P*=0.134). Among patients treated with chemotherapy, chemotherapy-related AEs occurred in 17 patients (39.5%) in the NIA group and 8 patients (42.1%) in the IA group, and the most common AEs was bone marrow suppression. There was no significant difference in chemotherapy-related AEs between the groups (*P*=0.099). Total treatment AEs occurred in 36 patients (83.7%) in the NIA group and 27 patients (75%) in the IA group, and there was no significant difference in total treatment AEs between the two groups (*P*=0.337).

## Discussion

This study evaluated the efficacy and safety of ICIs combined with antiangiogenic agents in elderly patients with advanced driver gene negative NSCLC. Retrospective analysis found that the addition of antiangiogenic agents to ICIs therapy did not provide significant clinical benefit, but the incidence of treatment interruptions due to irAEs was significantly reduced. Further studies found that elderly patients with advanced NSCLC in the PD-L1 high expression subgroup benefited from treatment with ICIs in combination with anti-angiogenic agents (PFS: *P*=0.017), suggesting PD-L1 ≥50% as a potential benefit marker in this population.

The median PFS in the NIA group in this study was 5.3 months, and the proportion of patients with combined chemotherapy was 88.4%, which was basically consistent with the 6.8 months of patients in the immune combined chemotherapy group in the IMpower150 study 13. The median OS in the NIA group was 30.9 months, similar to 27.9 months in the immune-combined chemotherapy group in the CAMEL Phase III study 10, but significantly different from 14.7 months in the IMpower150 final overall survival analysis. This may be related to the greater number of patients with PD-L1≥50% in our study (49.4% vs 25.2%). The ORR in the NIA group was 23.3%, which is different from 62.2% in the pembrolizumab+ chemotherapy group in KEYNOTE-407 11, which may be related to the higher number of patients with shorter PFS in this study. An FDA meta-analysis 16 evaluating immune monotherapy in elderly patients with NSCLC found a higher incidence of grade ≥3 adverse reactions in patients than in this study (49% vs 20.9%), which may be related to the shorter follow-up period in this study, in addition to the fact that the most common adverse reactions were the same in both studies.

Currently, there are three classes of anti-angiogenic drugs approved for the treatment of advanced NSCLC in China, including vascular endothelial growth factor inhibitor bevacizumab, small molecule multi-target tyrosine kinase inhibitor anlotinib, and recombinant human endostatin. Patients treated with bevacizumab or anlotinib were enrolled in this study. Several Phase III clinical studies 17-20 have confirmed the efficacy of bevacizumab combined with different platinum-based chemotherapy regimens in patients with advanced non-squamous NSCLC. The efficacy and safety of bevacizumab in elderly patients with advanced non-squamous NSCLC has also been confirmed in several studies 21, 22. In patients with advanced non-squamous NSCLC negative for driver mutations, IMpower150 and TASUKI-52 studies 13 confirmed that the addition of bevacizumab to immune-combination chemotherapy significantly improved patient outcomes, and subgroup analysis showed a similar benefit trend in older patients. In the ORIENT-31 study 23, sintilimab+ IBI305+ cisplatin and pemetrexed were generally effective and well tolerated compared to chemotherapy in patients with EGFR-mutated NSCLC who progressed after EGFR-TKI treatment.

Both ALTER0302 and ALTER0303 studies 15 confirmed the manageable efficacy and safety of anlotinib monotherapy in NSCLC patients. In the ALTER0303 study, which enrolled 28 elderly patients (age > 70 years), anlotinib significantly prolonged survival compared to placebo (mPFS: 11.2 months vs. 2.8 months, HR = 0.22, *P* = 0.003; mOS: 14.5 months vs. 6.3 months, HR = 0.34, *P* = 0.031) and was well tolerated. A retrospective study 24 found that anlotinib alone or in combination with ICIs is effective and well tolerated in the treatment of elderly patients with advanced NSCLC. A Phase Ib clinical study 14 has shown that anlotinib combined with sintilimab is a novel "chemo-free" treatment regimen for patients with advanced NSCLC due to its good efficacy and safety.

Elderly NSCLC patients were also included in some clinical studies involved bevacizumab and anlotinib. The therapeutic dose was similar to that of the overall population, and the tolerance was equal to that of non-elderly patients. And these two drugs have been included in the Consensus of Chinese Experts on Medical Treatment of Advanced Lung Cancer in the Elderly (2022 Edition) 25. Therefore, the incidence of immune-related adverse reactions in elderly patients was mainly assessed in this study. However, as elderly patients are often complicated with hypertension, coronary atherosclerotic heart disease and other cardiovascular and cerebrovascular diseases, so it is necessary to conduct more rigorous monitoring of AE that may occur during the use of angiogenesis inhibitors.

The results of several studies 26-30 have shown that the benefits of ICIs monotherapy or combination therapy are similar in elderly cancer patients and young cancer patients, and the report 31 also supports the safety of ICIs monotherapy in the elderly. Similarly, meta-analysis 32, 33 also found that elderly patients with advanced NSCLC who received ICIs could benefit from the PD-L1≥50% subgroup, but data on ICIs combined with antiangiogenic drugs in elderly lung cancer patients is rarely reported. Studies 34-37 have found that antiangiogenic drugs can block immunosuppressive signals by reversing vascular endothelial growth factor, promoting lymphocyte infiltration and migration and other pathways, and can also enhance immune efficacy by normalizing tumor blood vessels. Therefore, it is generally believed that ICIs combined with antiangiogenic drugs can have a synergistic effect.

This study found that elderly patients with advanced NSCLC in the PD-L1 high-expression subgroup could benefit from ICIs combined with anti-angiogenic drugs, and the IA group showed a safety advantage due to the lower proportion of combined chemotherapy compared to the NIA group. The incidence of AE after chemotherapy is higher in older patients than in younger patients, suggesting that chemotherapy is less well tolerated in the elderly and that chemotherapy risk assessment is needed before chemotherapy27 38, 39, and that "chemo-free" regimens have yielded positive results in some studies 14, 40. In addition, there is an unmet clinical need for effective strategies after immunotherapy resistance, and the treatment model of ICIs combined with anti-angiogenic drugs has shown great benefits 41-43.

Therefore, this study suggests that ICIs combined with anti-angiogenesis drugs is a safe and effective treatment for elderly NSCLC patients with PD-L1≥50%. PD-L1≥50% is a possible marker for patients who will benefit from ICIs combined with anti-angiogenesis therapy, and its significance in elderly patients needs to be further explored in clinical trials.

There are several limitations to this study: 1. Most of the patients enrolled were 65 to 75 years of age, which did not represent the elderly population well. 2. Because this was a retrospective study, the comprehensive geriatric score of patients could not be obtained, which reduced the reliability of the independent effect of combination drugs on prognosis. 3. Short follow-up time and the small number of patients with outcome events in the overall survival analysis may affect the results of the analysis. 4. The incidence of adverse events may have been reduced due to underreporting of adverse events. 5. Some patients received chemotherapy, radiotherapy and other treatments at the same time during the observation period, and the distribution of combined chemotherapy and chemotherapy frequency was not balanced between the two groups.

## Supplementary Material

Supplementary tables.

## Figures and Tables

**Table 1:** Baseline characteristics of 79 elderly advanced NSCLC patients

Variables	No. (%)		
	NIA group(n=43)	IA group(n=36)	*P*
**Age, years, mean** ±** SD**	69.9 ± 4.6	69.7 ± 3.8	0.917
**Histological type**			0.697
Non-squamous cell	22(51.2)	20(55.6)	
Squamous cell	21(48.8)	16(44.4)	
**PD-L1 expression**			0.559
Unmeasured or <1%	9(20.9)	7(19.4)	
1% - 50%	15(34.9)	9 (25.0)	
≥50%	19(44.2)	20(55.6)	
**Metastasis**			0.840
Brain	7(16.3)	6(16.7)	
Bone	17(39.5)	12(33.3)	
Others	19(44.2)	18(50)	
**History of tobacco use**			0.755
Never	17(39.5)	13(36.1)	
Former or current	26(60.5)	23(63.9)	
**Gender**			0.512
Female	6(14)	7(19.4)	
Male	37(86)	29(80.6)	
**ECOG status**			0.668
0 -1	34(79.1)	27(75)	
≥2	9(20.9)	9(25)	
**Line of therapy**			0.492
1	26(60.5)	19(52.8)	
≥2	17(39.5)	17(47.2)	
**Combined chemotherapy**			**<0.001**
No	5(11.6)	17(47.2)	
Yes	38(88.4)	19(52.8)	
**Combined radiotherapy**			0.210
No	33(76.7)	23(63.9)	
Yes	10(23.3)	13(36.1)	
**Metastatic number**			0.060
<3	26(60.5)	14(38.9)	
≥3	17(39.5)	22(61.1)	

**Table 2:** Univariate analysis for progression-free survival

Variables	Univariate analysis	
	HR (95%CI)	*P*
**Age, median (range, y)**	1.045(0.977-1.118)	0.196
**Histological type**		
Non-squamous cell	1	
Squamous cell	0.930(0.570-1.518)	0.772
**PD-L1 expression**		
Unmeasured or <1%	1	
1% - 50%	1.230(0.625-2.419)	0.549
≥50%	1.267(0.729-2.202)	0.401
**Metastasis**		
Brain	1	
Bone	0.964(0.494-1.880)	0.913
Others	0.642(0.362-1.138)	0.129
**History of tobacco use**		
Never	1	
Current or former	0.991(0.600-1.637)	0.973
**Gender**		
Female	1	
Male	0.800(0.391-1.638)	0.542
**ECOG status**		
0 -1	1	
≥2	0.820(0.438-1.537)	0.536
**Line of therapy**		
1	1	
≥2	1.003(0.611-1.649)	0.989
**Combined chemotherapy**		
No	1	
Yes	0.746(0.417-1.333)	0.322
**Combined radiotherapy**		
No	1	
Yes	0.750(0.438-1.284)	0.294
**Group**		
NIA	1	
IA	0.782(0.479-1.278)	0.326
**Metastatic number**		
<3	1	
≥3	1.161(0.711-1.896)	0.550

**Table 3:** Interaction analysis of patients on progression-free survival

Variables	B	SE	Wald	df	Sig.	Exp(B)	95%CI for Exp(B)	
							Lower	Upper
Group	0.527	0.340	2.393	1	0.122	1.693	0.869	3.301
PD-L1 expression	0.599	0.349	2.942	1	0.086	1.821	0.918	3.611
Interaction term	-1.573	0.517	9.263	1	**0.002**	0.207	0.075	0.571

The interaction term represents Group & PD-L1 expression.

**Table 4:** Univariate and multivariate analysis for overall survival

Variables	Univariate analysis Multivariate analysis			
	HR (95%CI)	*P*	HR (95%CI)	*P*
**Age, median (range, y)**	1.007(0.925-1.096)	0.875	/	/
**Histological type**				
Non-squamous cell	1		1	
Squamous cell	0.824(0.410-1.658)	0.588	0.538(0.225-1.287)	0.164
**PD-L1 expression**				
Unmeasured or <1%	1		1	
1% - 50%	**2.315(0.892-6.008)**	**0.084**	1.783(0.526-6.046)	0.353
≥ 50%	**3.051(1.371-6.790)**	**0.006**	2.560(0.851-7.702)	0.094
**Metastasis**				
Brain	1		1	
Bone	1.598(0.642-3.976)	0.313	0.917(0.333-2.524)	0.867
Others	1.136(0.515-2.505)	0.752	0.634(0.258-1.558)	0.321
**History of tobacco use**				
Never	1		/	/
Current or former	1.237(0.592-2.586)	0.571	/	
**Gender**				
Female	1		/	/
Male	0.962(0.370-2.502)	0.937	/	
**ECOG status**				
0 -1	1		1	
≥2	1.617(0.744-3.514)	0.225	0.861(0.350-2.117)	0.744
**Line of therapy**				
1	1		1	
≥2	0.773(0.379-1.580)	0.481	0.727(0.308-1.715)	0.466
**Combined chemotherapy**				
No	1		1	
Yes	**2.441(0.996-5.980)**	**0.051**	2.167(0.553-8.498)	0.267
**Combined radiotherapy**				
No	1		1	
Yes	**0.369(0.180-0.754)**	**0.060**	**5.046(2.016-12.632)**	**0.001**
**Group**				
NIA	1		/	/
IA	0.793(0.391-1.607)	0.520	/	
**Metastatic number**				
<3	1		/	/
≥3	0.833(0.413-1.680)	0.609	/	

**Table 5A:** Objective response rate

Best response	No. (%)		
	NIA group(n=43)	IA group(n=36)	*P*
CR	0(0.0)	1(2.7)	
PR	10(23.3)	10(27.8)	
SD	15(34.9)	13(36.1)	
PD	15(34.9)	11(30.6)	
Missing	3(7)	1(2.7)	
ORR, %	23.3	30.5	0.465

**Table 5B:** Objective response rate of patients in the subgroup of PD-L1 ≥ 50%

Best response	No. (%)		
	NIA group(n=19)	IA group(n=20)	*P*
CR	0(0.0)	1(5)	
PR	3(15.8)	6(30)	
SD	5(26.3)	8(40)	
PD	9(47.4)	4(20)	
Missing	2(10.5)	1(5)	
ORR, %	15.8	35	0.170

**Table 6:** Adverse events

AE	NIA group (n=43)		IA group (n=36)	
**Treatment-related AEs**				
Any grade	36(83.7)		27(75)	
≥3 grade	19(44.2)		9(25)	
**Immune-related AEs**	**Any grade**	**Grade≥3**	**Any grade**	**Grade≥3**
Myocarditis	1(2.3)	0(0.0)	0(0.0)	0(0.0)
Renal insufficiency	3(7.0)	3(7.0)	1(2.8)	0(0.0)
Rash	3(7.0)	1(2.3)	3(8.3)	1(2.8)
Diarrhoea	1(2.3)	0(0.0)	1(2.8)	1(2.8)
Pneumonitis	6(14.0)	4(9.3)	3(8.3)	1(2.8)
Hypophysitis	1(2.3)	0(0.0)	0(0.0)	0(0.0)
Hyperbilirubinemia	1(2.3)	1(2.3)	0(0.0)	0(0.0)
Itch	1(2.3)	0(0.0)	0(0.0)	0(0.0)
All	17(39.5)	9(20.9)	7(19.4)	3(8.3)
**Antiangiogenic drug-related AEs**	**Any grade**	**Grade≥3**	**Any grade**	**Grade≥3**
Hypertension	10(23.3)	1(2.3)	12(33.3)	3(8.3)
Thrombosis	1(2.3)	1(2.3)	1(2.8)	1(2.8)
Hyperlipidemia	5(11.6)	0(0.0)	4(11.1)	0(0.0)
Abnormal liver function	0(0.0)	0(0.0)	1(2.8)	0(0.0)
Gastrointestinal reaction	0(0.0)	0(0.0)	2(5.6)	0(0.0)
Bleeding	4(9.3)	2(4.7)	7(19.4)	1(2.8)
Protein urine	1(2.3)	0(0.0)	3(8.3)	0(0.0)
Rash	0(0.0)	0(0.0)	0(0.0)	0(0.0)
Hand-foot syndrome	0(0.0)	0(0.0)	0(0.0)	0(0.0)
Oral ulcer	1(2.3)	0(0.0)	2(5.6)	1(2.8)
All	19(44.2)	4(9.3)	22(61.1)	5(13.9)
**Chemotherapy-related AEs**	**Any grade**	**Grade≥3**	**Any grade**	**Grade≥3**
Bone marrow suppression	16(37.2)	8(21.1)	7(36.8)	3(15.8)
Gastrointestinal reaction	2(5.3)	2(5.3)	2(10.5)	0(0.0)
Abnormal liver and kidney function	1(2.6)	0(0.0)	0(0.0)	0(0.0)
Rash	0(0.0)	0(0.0)	0(0.0)	0(0.0)
Neurotoxicity	0(0.0)	0(0.0)	0(0.0)	0(0.0)
Alopecia	0(0.0)	0(0.0)	0(0.0)	0(0.0)
All	17(39.5)	9(20.9)	8(42.1)	3(15.8)

For patients, it was recorded as only once when more than 1 adverse event.

## Abbreviations

IA: immune checkpoint inhibitors plus angiogenesis inhibitors
NIA: immune checkpoint inhibitors without angiogenesis inhibitors
PD-L1: programmed cell death 1 ligand 1
ECOG: eastern cooperative oncology group
ICIs: immune checkpoint inhibitors
irAEs: immune-related adverse events
NR: not received
CIF: cumulative incidence function
SBRT: stereotactic ablative radiotherapy
