# Supplementary material for: Efficacy and Safety Analysis of Immune Checkpoint Inhibitors plus Angiogenesis Inhibitors for the Treatment of Advanced Driver-negative NSCLC in Elderly Patients: A Retrospective Study
Source: J Cancer. 2023 Jun 4;14(9):1623–34. doi: 10.7150/jca.83719 (PMC10266243; doi:10.7150/jca.83719)
Supplement: Supplementary file 1 — Supplementary tables. [file jcav14p1623s1.pdf]

Table S1. Other characteristics of 79 elderly advanced NSCLC patients

Variables	No. (%)		
	NIA group (n=43)	IA group (n=36)	*P*
**Age**			
≤70	28(65.1)	22(61.1)	0.713
＞70	15(34.9)	14(38.9)	
**ICIs**			0.113
Sintilimab	12(27.9)	20(55.6)	
Camrelizumab	7(16.3)	5(13.9)	
Pembrolizumab	10(23.3)	4(11.1)	
Toripalimab	4(9.3)	0(0.0)	
Tislelizumab	7(16.3)	6(16.7)	
Durvalumab	2(4.7)	1(2.8)	
Atezolizumab	1(2.3)	0(0.0)	
**Cycles of immunotherapy**			0.333
≥6	18(41.9)	19(52.8)	
2~5	25(58.1)	17(47.2)	
**Therapy duration，month**	5±4.4	5±3.3	0.565
**Antiangiogenic agents**			/
Anlotinib	/	15(41.7)	
Bevacizumab	/	15(41.7)	
Endostatin	/	6(16.7)	
**Combined chemotherapy**			**＜0.001**
Yes	38(88.4)	19(52.8)	
No	5(11.6)	17(47.2)	
**Cycles of combined chemotherapy**			**0.007**
≥ 4	25(58.1)	10(27.8)	
＜4	18(41.9)	26(72.2)	
**Radiotherapy site**			0.743
Primary lesion	3(7)	5(13.9)	
Brain	3(7)	3（8.3)	
Bone	2(4.7)	3(8.3)	
Liver	0(0.0)	0(0.0)	
Other sites	2(4.7)	2(5.6)	
None	33(76.7)	23(63.9)	
**Radiotherapy technology**			0.338
SBRT	3(7)	2(5.6)	
Not SBRT	7(16.3)	11(30.6)	
None	33(76.7)	23(63.9)	

Table S2. Tumor burdens of all patients

Characteristics	Patients [n (%)]
n	79(100)
**Clinical T Stage**	
T1-T3	55(69.6)
T4	20(25.3)
Missing	4(5.1)
**Clinical N Stage**	
N0-N2	69(87.3)
N3	8(10.1)
Missing	2(2.5)
**Cancer type**	
Primary metastatic disease	45(57)
Recurrent disease	34(43)
**Site of metastases or recurrence**	
Distant	48(60.8)
Locoregional	31(39.2)
**No. of involved organs**	
0	31(39.2)
1	37(46.8)
⩾2	11(13.9)
**Site of metastases**	
Brain	14(17.7)
Bone	27(34.2)
Liver	4(5.1)
Other sites	34(43)

If there are more than 2 metastatic organs for patients, the organ of the worse prognosis is regarded as “site of metastases”. Prognosis: Brain > Bone > Liver > Other site.

Table S3. Details of radiotherapy and chemotherapy in patients combined with radiotherapy during PFS period

No.	SBRT	Treatment Site	Dose (Gy)	Fractions	Chemo-therapy	Prog-ression	Local failure	Distant metastasis	Site
1	No	Brain	30	10	Yes	Yes	No	Yes	Prostate gland Rectum、Ureter
2	Yes	Left upper lobe	50	5	No	Yes	No	Yes	Bone
3	No	Right T12 Paraspinal	39	13	Yes	Yes	No	No	-
4	Yes	Right occipital lobe	27	3	Yes	Yes	No	No	-
5	No	parietal bone	55	20	Yes	Yes	Yes	No	Adrenal gland
6	No	Abdominal wall	39	13	Yes	Yes	No	Yes	Adrenal gland
7	No	Right hilar	40	20	Yes	Yes	No	Yes	Bone
8	Yes	Left lung	18	3	No	Yes	No	No	-
9	No	Right lung	39	13	Yes	Yes	No	No	-
10	No	Brain	40	20	Yes	Yes	Yes	Yes	Liver Spleen Bone
11	No	Lumbar vertebra	40	20	No	No	-	-	-
12	No	Right upper lobe Lymph node	40	20	Yes	No	-	-	-
13	No	Brain	30	10	No	Yes	No	-	-
14	No	Left hilar	60	30	No	Yes	No	Yes	Bone
15	Yes	Left lower lobe	50	5	Yes	Yes	Yes	No	-
16	No	Lymph node	60	30	No	Yes	-	-	-
17	No	Thoracic vertebra	18	6	No	Yes	No	Yes	Brain
18	No	Lymph node	30	10	Yes	Yes	No	Yes	Bone
19	No	Right lung	50	25	No	Yes	Yes	No	-
20	No	Brain	40	20	Yes	Yes	No	Yes	Bone
21	No	Tumor bed Right hilar Lymph node	50	25	No	Yes	No	Yes	Brain
22	No	Right lung Right T4	50	20	No	No	-	-	-
23	Yes	Brain	40	8	No	No	-	-	-
